# Alcohol use disorders and risk of Parkinson’s disease: findings from a Swedish national cohort study 1972–2008

**DOI:** 10.1186/1471-2377-13-190

**Published:** 2013-12-05

**Authors:** Anna-Karin Eriksson, Sofia Löfving, Russell C Callaghan, Peter Allebeck

**Affiliations:** 1 Department of Public Health Sciences, Division of Social Medicine, Karolinska Institutet, Stockholm, Sweden; 2 Northern Medical Program, University of British Columbia, Prince George, British Columbia, Canada

**Keywords:** Alcohol, Parkinson’s disease, Cohort, Longitudinal, Epidemiology, Register

## Abstract

**Background:**

Alcohol has been suggested to be either protective of, or not associated with Parkinson’s disease (PD). However, experimental animal studies indicate that chronic heavy alcohol consumption may have dopamine neurotoxic effects relevant for PD. We studied the association between diagnosed alcohol use disorders and PD.

**Methods:**

All individuals in Sweden admitted with a diagnosis of an alcohol use disorder or appendicitis (reference group) between January 1, 1972 and December 31, 2008 were identified through the Swedish National Inpatient Register, and followed for up to 37 years for a diagnosis of PD. We estimated hazard ratios (HR) with 95% confidence intervals (CI) and adjusted for age and sex.

**Results:**

We found 1,741 (0.3%) cases of PD in the cohort of 602,930 individuals, 1,083 (0.4%) among those admitted with an alcohol use disorder and 658 (0.2%) of the individuals admitted with appendicitis. The mean follow-up time was 13.6 and 17.1 years, respectively. The HR for PD associated with an alcohol use disorder was 1.38 (CI 1.25-1.53) adjusted for age and sex. When the risk was estimated in age groups for first hospital admission with PD the highest risk was observed in the lowest age group, **≤**44, HR 2.39 (0.96-5.93), adjusted for age at exposure and sex.

**Conclusions:**

A history of an alcohol use disorder conferred an increased risk of admission with a diagnosis of Parkinson’s disease in both women and men. In particular, the risk seemed higher at lower ages of first admission with Parkinson’s disease.

## Background

Little is known about the aetiology of Parkinson’s disease (PD), which is the second most common neurodegenerative disorder following Alzheimer’s disease. It is believed that major gene mutations cause only a small proportion of the cases and that environmental factors are involved. Occupational exposures such as pesticides and herbicides seem to increase the risk for PD, and possibly lead to damage of dopaminergic cells [1,2].

Alcohol has been suggested to protect from neurodegeneration and vascular disease although the mechanisms are not clear [3]. For instance flavonoids that can be found in red wine, might have neuro [4] and vascular protective effects [5]. One review from 2005 came to the conclusion that there was some evidence for a decreased risk of PD associated with alcohol consumption, although most studies did not reach statistical significance [3]. Another review from 2011 states the epidemiological evidence on these associations to be limited [6]. Even though there is no association between lower consumption of alcohol and PD, or a modest protective effect, it is still possible that chronic heavy consumption of alcohol could have dopamine neurotoxic effects relevant for PD, as shown in an experimental animal study by Gilman et al. [7].

Most previous studies on alcohol consumption and the risk of PD have used cross-sectional designs, including self-report of low to moderate alcohol consumption. At this time, most studies have not addressed adequately the risk of PD among individuals with heavy alcohol use or those who would qualify for alcohol abuse or dependence diagnoses. Also, studies investigating the prospective association between alcohol use disorders and PD which takes into account the long latency period for developing PD have not been performed. To our knowledge, only one study has investigated the association between diagnosed alcohol use disorders and PD [8]. This study used the General Practice Research Database in the United Kingdom and found no association, although had a limited follow-up period.

The aim of this study was to investigate the influence of alcohol use disorders on the risk for developing PD in a national-wide study with a long follow-up period. We prospectively studied in a cohort from the Swedish National Inpatient Register, the association between a diagnosis of an alcohol use disorder and a diagnosis of PD up to 37 years later and compared with a population-proxy group.

## Methods

### Study population and exclusion criteria

We included all men and women in Sweden who were hospitalized with either a diagnosis of an alcohol use disorder or appendicitis between January 1, 1972 and December 31, 2008, identified through the Swedish National Inpatient Register. This register is kept by the National Board of Health and Welfare and covers the entire Swedish population.

The individuals with an alcohol use disorder (exposed group), and the individuals with appendicitis (population-proxy reference group), were followed with regard to a diagnosis of PD (see disease classifications below), recorded in the Swedish National Inpatient Register up to 2008, i.e. a maximum follow-up of 37 years. Information about deaths was obtained from the National Cause of Death Register through record linkage of the cohort using the individually unique personal identification numbers. Individuals were excluded if they belonged to one or more of the following groups: 1) had been hospitalized with a diagnosis of PD prior to or concurrent with admission for an alcohol use disorder or appendicitis, n = 630; 2) had been hospitalized with a diagnosis of Parkinsonism (diagnostic codes ICD-8: 342.08 and 342.09, ICD-9: 332B and 333A-X, or ICD-10: G21-G26) prior to or concurrent with admission for an alcohol use disorder or appendicitis, n = 146; 3) had been hospitalized with a diagnose for substance abuse other than alcohol, i.e. admitted with ICD-8, ICD-9 and ICD-10 amphetamine-related codes: 304.60, 304E, 969H, F15.0-F15.9, T43.6, n = 7083; cocaine-related codes: 304.40, 304C, F14, T40.5, n = 441; or opioid-related codes: 304.00, 304.10, 304A, 965A, F11, T40.0, T41.0, T40.2, n = 2642. After exclusions, the study comprised 276,527 individuals with an alcohol use disorder (73,794 women and 202,733 men) and 326,403 individuals with appendicitis (149,233 women and 177,178 men).

The study was approved by the Stockholm Regional Ethical Review Board, Sweden.

### Classification of alcohol exposure

Each individual was considered to be exposed from the time of his or her first admission with a diagnosis of an alcohol use disorder recorded in the Swedish National Inpatient Register during the study period. Survival time was calculated as the interval between this date and the date of first admission with PD, administrative censoring on 31 December 2008, or as recorded in the National Cause of Death Register, whichever came first. The criteria for assignment to the cohort with alcohol diagnoses were: ICD-8: 291.00-.99 (Alcohol psychosis), 303.00-.99 (Alcoholism); ICD-9: 291A-X (Alcohol psychoses), 303 (Alcohol addiction), 305A (Misuse of alcohol), 980A-X (Toxic effect of alcohol); ICD-10: F10.0-9 (Psychiatric disorders caused by alcohol), F10.0-.9 (Psychiatric and behavioral disorders caused by alcohol), T51.0-9 (Toxic effect of alcohol), X45 (Unintentional intoxication with and exposure to alcohol).

### Classification of PD

First admission with a diagnosis of PD registered in the Swedish National Inpatient Register was defined according to the Swedish version of the eighth (ICD-8), ninth (ICD-9) and tenth (ICD-10) revisions of the WHO International Classification of Diseases, with the following codes: ICD-8: 342.00; ICD-9: 332A; and ICD-10: G20. The accuracy of the PD diagnoses in the Swedish National Inpatient Register has been reported to be 70.8% [9]. The cases were identified by the main and up to seven contributory diagnoses. To be classified as a case, the date of first admission with PD had to be registered at least 1 year later than the date for admission for an alcohol use disorder or appendicitis. In the cohort, 9,761 individuals had been hospitalized for both an alcohol use disorder and appendicitis at different occasions. These individuals were analysed as exposed to an alcohol use disorder regardless of which diagnose that appeared first in time.

### Population-proxy reference group

Individuals with appendicitis served as the population-proxy comparison group, given that appendicitis: 1) is a relatively frequent reason for admission to hospital; 2) does not appear to be related to PD, alcohol use disorders or other drug disorders; 3) has been used successfully as a population-proxy reference group in other studies [10]; and 4) groups drawn from inpatient medical records have shown to have an incidence of PD not different from that of the general population [11]. Inclusion criteria for this cohort consisted of: 1) a diagnosis of an appendicitis-related condition at their index admission (ICD-8 or ICD-9 codes 540–542; or ICD-10 codes K35-K37 identified by the main and up to seven contributory diagnoses); 2) no prior, concurrent, or subsequent indication of any alcohol- or other drug-use diagnoses.

### Data analysis

Cox regression analysis was used to calculate hazard ratios (HR) with 95% confidence intervals (CI) for the association between a diagnosis of an alcohol use disorder and later PD. Outcome was first admission with a diagnosis of PD. Also, we assessed the risk by age at first admission for PD. We ran the analyses both with and without adjustments for sex and age at first admission for an alcohol use disorder or appendicitis. All analyses were performed in SAS version 9.2 (SAS Institute, Cary, NC, USA).

## Results

We identified 1,741 cases of PD (0.3%) in the total cohort of 602,930 individuals in the Swedish National Inpatient Register (Table 1). Of those who had been hospitalized with an alcohol use disorder, 1,083 (0.4%) had been admitted with PD, 208 women (0.3%) and 875 men (0.4%). In the population-proxy reference group, i.e. the individuals admitted with a diagnosis of appendicitis, 658 (0.2%) were later admitted with PD, 271 women (0.2%) and 387 men (0.2%). For both women and men, the greatest proportion of cases occurred in individuals 60 years and older at the time of exposure measurement. The mean follow-up time was 13.6 years for the group with alcohol use disorders and the mean age at exposure 43.0 years. In the group with appendicitis the mean follow-up time was 17.1 years and the mean age at exposure 30.0 years.

**Table 1:** Number of individuals with a diagnosis of an alcohol use disorder or appendicitis (n = 602,930) in the Swedish National Inpatient Register 1972–2008, distributed by age and sex

	**Alcohol use disorders (n = 276,527)**		**Parkinson’s disease among individuals with alcohol use disorders (n = 1,083)**		**Appendicitis (n = 326,403)**		**Parkinson’s disease among individuals with appendicitis (n = 658)**	
	**Women (%)**	**Men (%)**	**Women (%)**	**Men (%)**	**Women (%)**	**Men (%)**	**Women (%)**	**Men (%)**
**Total**	73,794 (100)	202,733 (100)	208 (100)	875 (100)	149,233 (100)	177,178 (100)	271 (100)	387 (100)
**Age**								
≤29	24,691 (33.5)	43,737 (21.6)	0	0	89,676 (60.1)	106,762 (60.3)	2 (0.7)	3 (0.8)
30 - 44	20,542 (27.8)	61,904 (30.5)	4 (1.9)	10 (1.1)	26,653 (17.9)	36,070 (20.4)	2 (0.7)	3 (0.8)
45 - 59	18,539 (25.1)	60,109 (29.7)	28 (13.5)	91 (10.4)	16,996 (11.4)	18,952 (10.7)	16 (5.9)	37 (9.6)
60 - 74	7,989 (10.8)	31,377 (15.5)	103 (49.5)	456 (52.1)	11,081 (7.4)	11,527 (6.5)	93 (34.3)	133 (34.4)
≥75	2,033 (2.8)	5,606 (2.8)	73 (35.1)	318 (36.3)	4,827 (3.3)	3,859 (2.2)	158 (58.3)	211 (54.5)

The crude HR for PD was 2.57 (2.32-2.82) in the individuals with an alcohol use disorder compared to the reference group (Table 2). Adjustment for sex did not substantially alter the estimate. However, when age was adjusted for the HR was 1.52 (1.38-1.67). The final model including sex and age resulted in a HR of 1.38 (1.25-1.53). When analysed separately, the HR in women was 1.64 (1.37-1.97) and in men 1.28 (1.14-1.44) adjusted for age (Table 3). When stratified by age groups at first hospital admission for PD, the highest HR was found in those with age ≤44, HR 2.39 (0.96-5.93) (Table 4).

**Table 2:** HRs and 95% CIs for the association between alcohol use disorders and Parkinson’s disease in the Swedish National Inpatient Register 1972-2008, n = 602,930

		**Total population**		** Parkinson’s disease**		
		***n***	***n***	**HR**^**a**^	**(95% CI)**	***p-value***^**b**^
Appendicitis	Reference	326,403	658	1		
Alcohol	Model 1^c^	276,527	1,086	2.57	(2.32–2.82)	<.0001
	Model 2			1.52	(1.38–1.67)	<.0001
	Model 3			2.42	(2.19–2.67)	<.0001
	Model 4			1.38	(1.25–1.53)	<.0001

^a^Abbreviations: HRs, hazard ratios.

^b^Tested with 95% significant level.

^c^Model 1 crude.

Model 2 adjusted for age at exposure.

Model 3 adjusted for sex.

Model 4 adjusted for age at exposure and sex.

**Table 3:** HRs and 95% CIs for the association between alcohol use disorders and Parkinson’s disease in women and men in the Swedish National Inpatient Register 1972-2008, n = 602,930

		** Women**					** Men**				
		**Total**	**Parkinson’s disease**				**Total**	**Parkinson’s disease**			
		***n***	***n***	**HR**^**a**^	**(95% CI)**	***p-value***^**b**^	***n***	***n***	**HR**^**a**^	**(95% CI)**	***p-value***^**b**^
Appendicitis	Reference	149,233	271	1			177,170	387	1		
Alcohol	Model 1^c^	73,794	208	2.31	(1.92–2.77)	<.0001	202,733	878	2.48	(2.20–2.79)	<.0001
	Model 2			1.64	(1.37–1.97)	<.0001			1.28	(1.14–1.44)	<.0001

^a^Abbreviations: HRs, hazard ratios.

^b^Tested with 95% significant level.

^c^Model 1 crude.

Model 2 adjusted for age at exposure.

**Table 4:** HRs and 95% CIs for the association between alcohol use disorders and Parkinson’s disease distributed by age of first admission with Parkinson’s disease in the Swedish National Inpatient Register 1972-2008, n = 602,930

	**Total population**	** ≤44**				** 45-59**				** 60-74**				** ≥75**			
	***n***	***n***	**HR**^**a**^	**(95% CI)**	***p-value***^***b***^	***n***	**HR**^**a**^	**(95% CI)**	***p-value***^***b***^	***n***	**HR**^**a**^	**(95% CI)**	***p-value***^***b***^	***n***	**HR**^**a**^	**(95% CI)**	***p-value***^***b***^
Appendicitis ref	326,403	10	1			53	1			226	1			369	1		
Alcohol model 1^c^	276,527	14	2.71	(1.20–6.10)	0.016	119	3.58	(2.59–4.94)	0.0001	559	3.78	(3.24–4.41)	<.0001	391	1.67	(1.45–1.93)	<.0001
Model 2			2.41	(0.98–5.90)	0.056		1.94	(1.38–2.72)	<.0001		2.00	(1.71–2.34)	<.0001		1.03	(0.89–1.18)	0.732
Model 3			2.69	(1.17–6.12)	0.020		3.37	(2.42–4.69)	<.0001		3.55	(3.03–4.16)	<.0001		1.60	(1.38–1.85)	<.0001
Model 4			2.39	(0.96–5.93)	0.060		1.84	(1.30–2.60)	0.0006		1.84	(1.58–2.18)	<.0001		0.91	(0.78–1.06)	0.226

^a^Abbreviations: HRs, hazard ratios.

^b^Tested with 95% significant level.

^c^Model 1 crude.

Model 2 adjusted for age at exposure.

Model 3 adjusted for sex.

Model 4 adjusted for age at exposure and sex.

## Discussion

In this cohort study, heavy alcohol consumption as indicated by an admission with an alcohol use disorder conferred an increased risk of PD in both women and men. When the HRs were calculated for age groups of first admission with PD and adjusted for age at exposure and sex, the influence of alcohol consumption seemed to be greater at younger PD ages.

Given that PD has been linked to environmental factors, it is possible that factors associated with an alcohol-related diagnosis, such as poor nutrition, lower socioeconomic status or occupational exposure to PD-related toxins, might confound the relation between alcohol use disorders and PD. In this study we did not have the opportunity to control for potential confounders other than age. At the same time, very little is known about which risk factors are influencing the risk for PD. Notably, we did not adjust for smoking which is one of few factors that has consistently been found to decrease the risk for PD [11]. Thus, since there is a covariation between alcohol consumption and smoking, the true risk for PD associated with alcohol consumption in this study might be higher than our findings. When adjusting the estimates for age, the HRs were reduced. This can be explained by the difference in mean age of admission for the two exposure groups in our study; the mean age of first admission with an alcohol use disorder was higher. I.e. the group with alcohol use disorders was admitted to inpatient care later in life, although their alcohol abuse most probably have started much earlier, compared to the group with an appendicitis, for which patients are admitted immediately, and at a younger age.

According to our knowledge only one other study has reported an increased risk of PD associated with alcohol consumption [12]. Reviews compiling the current knowledge on risk factors for PD [3,6] report a modest risk reduction of PD due to alcohol consumption, or no association*.* The previous literature is very diverse with regard to measurement of alcohol consumption, reference groups, study populations, ways of diagnosing PD and case identification. Also, there may have been no possibility of separating diagnoses of Parkinsonism from PD, or to study individuals with extreme alcohol consumption. The previous studies differ from the present study with regard to exposure measurement. We used inpatient diagnoses of alcohol use disorders and our comparison group was the individuals not having been hospitalized for an alcohol use disorder, however, could have consumed alcohol in different quantities (i.e. they were probably not abstainers). Given that it is likely that the control group also consume some amounts of alcohol, this would imply that we might have underestimated the risk.

There are good reasons to think that excessive consumption of alcohol could increase the risk of PD. The direct effects of alcohol include dopamine release [13]. However, the brain is very sensitive to alcohol and chronic consumption can cause severe brain damage [14]. It has been demonstrated that chronic heavy alcohol consumption may have dopamine neurotoxic effects, damaging the central nervous system extensively, including both regions of the cerebral cortex and cerebellum, and the substantia nigra or its dopaminergic projections to the putamen, or both [7]. It is also possible that alcohol contributes to oxidative stress that may affect the brain [15,16]. However, we can only speculate about why the influence of alcohol seemed to be greater at younger PD ages. It could be due to that the neurotoxic dopaminergic effects of alcohol might be more dramatically different in the group exposed to alcohol at a younger age, when the control group has no or very little dopaminergic deficits.

The strengths of this study include its prospective and population-based design and that we study alcohol exposure by diagnoses of alcohol use disorders, i.e. we are measuring excessive consumption and are also avoiding recall bias from retrospective self-report. The majority of the previous studies in this area have utilized case–control designs, which might introduce recall bias of exposure levels [17]. Present consumption is likely to influence the report of past consumption, and a recent study found that individuals with PD decreased their alcohol intake at the time of diagnosis [18]. Further, ours is a relatively large study with a follow-up period of a maximum of 37 years. In addition, we were able to exclude individuals that had been hospitalized for other drug use, and individuals with Parkinsonism and PD prior or current to exposure.

Some limitations in our study should be acknowledged. The cases of PD were derived from the Swedish National Inpatient Register, a data source that has been shown to be valid for epidemiological studies on PD aetiology [19]. The accuracy of PD diagnoses in the Swedish National Inpatient Register has been evaluated against a screening study of 35,000 twins, and the positive predictive value for PD diagnoses was found to be 70, 8%. When the analyses were restricted to only primary diagnoses the positive predictive value was further improved to 83, 0%, however this leads to reduced sensitivity [19]. Register information about outpatient care in hospital-based clinics in Sweden are available only from 2001. While most treatment for PD occurs in outpatient settings, our outcome measure captured only the PD cases admitted to hospital. Nonetheless, prior research has shown that a majority of individuals with PD are admitted to hospital at least once within six years of their initial diagnosis [20]. It may be mentioned that it is possible that the group exposed to alcohol use disorders is more frequently admitted to hospital care and thereby more likely to be assessed for neurological disorders such as PD compared to the reference group of individuals with appendicitis. In that case we could have over-estimated the risk for PD associated with alcohol exposure. However, the opposite is also possible and more plausible, i.e. that the group with alcohol-related disorders is underrepresented in inpatient care.

We cannot exactly establish the point in time for a PD diagnosis as we are using inpatient data, since most PD cases are first detected and diagnosed in outpatient care. However, we have excluded inpatient cases of PD and Parkinsonism before, and at the time of admission with an alcohol related disorder. Also, PD is a typical age-related disease, usually occurring only after 50 years of age, while severe alcohol problems usually start much earlier in the life-span. As a result, reversed causality in our findings is unlikely. Also, we excluded cases of PD within one year after first admission with an alcohol use disorder. Extending this period to up to five years did not substantially change the estimates (data not shown)*.*

## Conclusions

We found an increased risk of admission with a diagnosis of PD for both women and men with a history of an alcohol use disorder. Given the high level of excessive alcohol use in the population, an increased risk of a serious neurodegenerative disease like PD is of public health importance.

## Competing interests

The authors declare that they have no competing interests.

## Authors’ contributions

A-KE researched data and wrote the manuscript. SL researched data and reviewed the manuscript. RCC and PA designed the study and reviewed the manuscript. All authors read and approved the final manuscript.

## Pre-publication history

The pre-publication history for this paper can be accessed here:

http://www.biomedcentral.com/1471-2377/13/190/prepub
